# antipsychotics and metabolic syndrome

**DOI:** 10.1192/j.eurpsy.2022.1923

**Published:** 2022-09-01

**Authors:** R. Jomli, H. Jemli, U. Ouali, A. Ouertani, S. Madouri

**Affiliations:** 1 Razi Hospital, Psychiatry A, manouba, Tunisia; 2 university of tunis elmanar, Faculty Of Medicine Of Tunis, manouba, Tunisia

**Keywords:** psychoeducation, obesity, antipsychotic

## Abstract

**Introduction:**

Patients treated for chronic mental disorders and who receive atypical antipsychotics are in most cases at risk of gaining weight, the excess of which is complicated in the long term by metabolic syndrome (MS). The management of these patients is effective if it includes Therapeutic Education.

**Objectives:**

Describe the therapeutic education program developed for patients on antipsychotics who have metabolic syndrome

**Methods:**

In this work, we present the educational program that we have developed for patients undergoing psychiatric treatment with atypical antipsychotics, who have been stabilized for at least 3 months and who suffer from SM.

**Results:**

It is a program that starts with the inclusion consultation and educational diagnosis with the first step of clinical (weight, abdominal perimeter and BMI), biological (blood sugar, HbA1C, cholesterol, HDL, triglycerides) and psychometric (SF12, MAQR, food and physical activity diary) assessments. Our initial program includes 6 sessions and 2 maintenance sessions at 1 month and 3 months after the 6th session. The objectives were divided between information about DM, motivation to eat a balanced diet, physical activity and improvement of quality of life. We also included stress management and positive psychology activities. Assessments are repeated at the end of the initial program and at the last maintenance session.

**Conclusions:**

Our program was developed according to the Geneva therapeutic education recommendations. We plan to apply it to groups of patients in our department

**Disclosure:**

No significant relationships.

